# Geographic disparities, determinants, and temporal changes in the prevalence of pre-diabetes in Florida

**DOI:** 10.7717/peerj.10443

**Published:** 2021-01-13

**Authors:** Jennifer Lord, Shamarial Roberson, Agricola Odoi

**Affiliations:** 1 Biomedical and Diagnostic Sciences, College of Veterinary Medicine, The University of Tennessee, Knoxville, TN, United States of America; 2 Florida Department of Health, Tallahassee, FL, United States of America

**Keywords:** Pre-diabetes, Spatial epidemiology, Geographic disparities, Florida, Geographical Information Systems, Spatial Scan Statistics, Tango’s Flexible Scan Statistics

## Abstract

**Background:**

Left unchecked, pre-diabetes progresses to diabetes and its complications that are important health burdens in the United States. There is evidence of geographic disparities in the condition with some areas having a significantly high risks of the condition and its risk factors. Identifying these disparities, their determinants, and changes in burden are useful for guiding control programs and stopping the progression of pre-diabetes to diabetes. Therefore, the objectives of this study were to investigate geographic disparities of pre-diabetes prevalence in Florida, identify predictors of the observed spatial patterns, as well as changes in disease burden between 2013 and 2016.

**Methods:**

The 2013 and 2016 Behavioral Risk Factor Surveillance System data were obtained from the Florida Department of Health. Counties with significant changes in the prevalence of the condition between 2013 and 2016 were identified using tests for equality of proportions adjusted for multiple comparisons using the Simes method. Flexible scan statistics were used to identify significant high prevalence geographic clusters. Multivariable regression models were used to identify determinants of county-level pre-diabetes prevalence.

**Results:**

The state-wide age-adjusted prevalence of pre-diabetes increased significantly (*p* ≤ 0.05) from 8.0% in 2013 to 10.5% in 2016 with 72% (48/67) of the counties reporting statistically significant increases. Significant local geographic hotspots were identified. High prevalence of pre-diabetes tended to occur in counties with high proportions of non-Hispanic black population, low median household income, and low proportion of the population without health insurance coverage.

**Conclusions:**

Geographic disparities of pre-diabetes continues to exist in Florida with most counties reporting significant increases in prevalence between 2013 and 2016. These findings are critical for guiding health planning, resource allocation and intervention programs.

## Introduction

People with pre-diabetes are considered to be at higher risk of developing diabetes and subsequent complications than those that do not have the condition. In 2015, an estimated 33.9% of adults had pre-diabetes in the United States, and the percentage of pre-diabetics among seniors aged 65 years and older was estimated to be 48.3% (CDC, 2017). Lifestyle modifications targeting physical activity level and obesity are among the most important interventions used to prevent pre-diabetes and potential progression to diabetes (Tabák et al., 2012). However, many adults with pre-diabetes in the United States remain undiagnosed. Among those with glycemic parameters consistent with the condition, only 11.6% had been diagnosed by a health professional (CDC, 2017).

Like many other chronic conditions, there are geographic disparities in the prevalence of pre-diabetes. Previous studies investigating these disparities have been limited in that they have been descriptive in nature and very few have used rigorous statistical/epidemiological spatial cluster investigation techniques to identify these disparities and disease hotspots at sub-state levels and yet findings from such investigations are critically important for guiding health planning and resource allocation. Moreover, many of them have focused on diabetes and not pre-diabetes. However, one of our previous studies, that used rigorous statistical/epidemiological cluster investigation techniques and data from the 2013 Florida Behavioral Risk Factor Surveillance System (BRFSS), detected multiple high-prevalence clusters of both pre-diabetes and diabetes across the state (Lord, Roberson & Odoi, 2020). Moreover, that study also found that predictors of pre-diabetes and diabetes at the individual level differed based on whether individuals lived inside or outside a hotspot county. This suggests that detailed investigations at sub-state levels, using rigorous statistical/epidemiolocal approaches, are critically important to guide needs-based planning, resources allocation, service provision, prevention and control strategies as well as policy. Unfortunately, similar analyses of pre-diabetes distribution and its determinants are currently lacking in the published literature. Ongoing monitoring and rigorous assessment of the spatial distribution of pre-diabetes, as well as identifying determinants of observed disparities using rigorous epidemiological approaches, are necessary to guide evidence-based health planning at the sub-state levels. Therefore, the objectives of this study were to: (1) investigate spatial patterns and clustering of pre-diabetes prevalence at the county level in Florida in 2016; (2) investigate county-level predictors of the spatial distribution of pre-diabetes, and (3) identify temporal changes, if any, in the geographic distribution of pre-diabetes between 2013 and 2016.

## Material and Methods

### Ethics approval and consent to participate

This study was approved by the University of Tennessee, Knoxville Institutional Review Board (Number: UTK IRB-19-05440-XM).

### Study area

**Figure 1:**
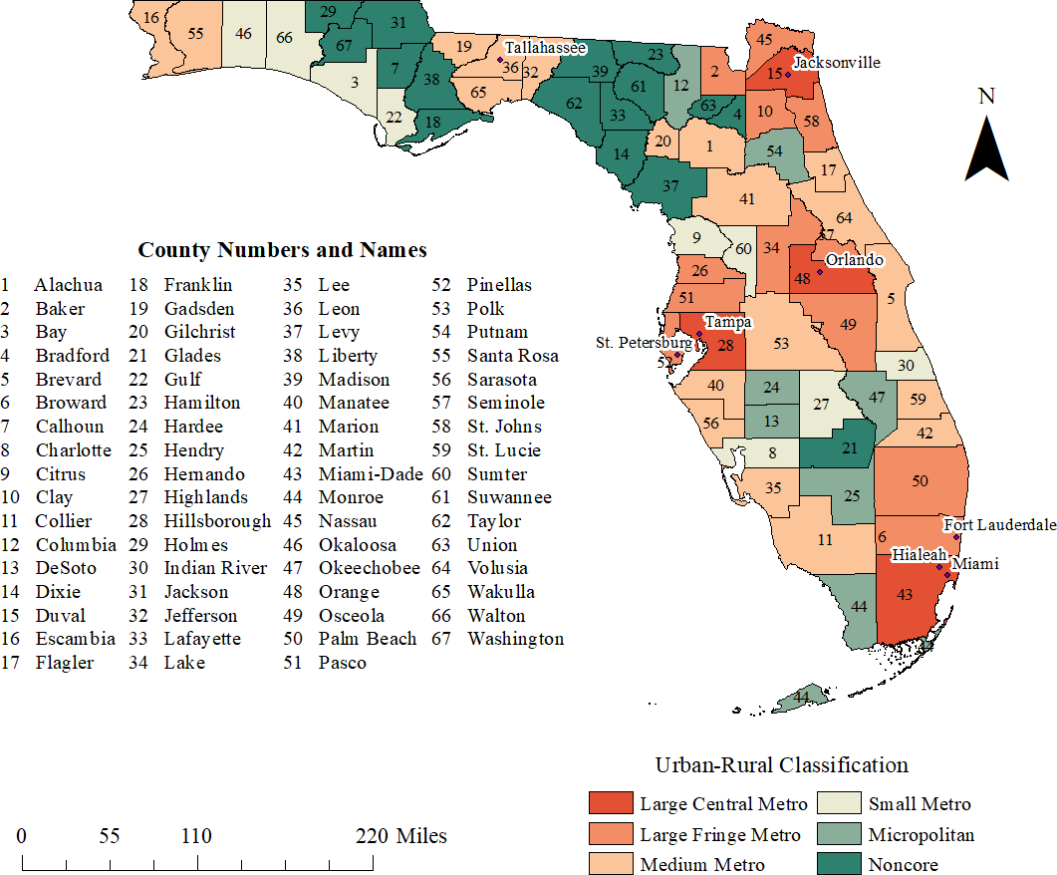
Urban-rural classification and geographic distribution of counties and major cities in the state of Florida, USA.

This study was performed in Florida, which has 67 counties. Based on the American Community Survey (ACS) 5-year estimates for 2012–2016, the state had a population of 19.9 million people (US Census Bureau, 2016a). The most populated county was Miami-Dade, with 2.66 million people, and the least populated was Liberty County, with 8,285 people (Fig. 1).

### Data sources and data preparation

This retrospective study used secondary data and hence did not involve obtaining consent. A list of data sources used in the study is summarized in Table 1. The county-level cartographic boundary file, used as foundation data for mapping, was obtained from the United States Census Bureau TIGER Geodatabase (US Census Bureau, 2016b).

**Table 1:** Data sources and variables used in the study of geographic disparities, determinants and temporal changes in prevalence of pre-diabetes in Florida.

**Source**	**Data obtained**
United States Census Bureau TIGER Geodatabase	County-level cartographic boundary shapefile
2013 and 2016 Florida Behavioral Risk Factor Surveillance System (BRFSS)	Respondent prediabetes status (self-reported) Respondent diabetes status (self-reported) Respondent’s county of residence Age of respondent Body mass index of respondent (BMI) Physical activity level of respondent Respondent arthritis status Respondent disability status Respondent health insurance status
Li et al. (2014)	2010 US Standard Population for age adjustment
United States Census Bureau American Community Survey 5-year estimates (2012–2016 and 2009–2013)	Median household income Percent of the population 16 years and older who are unemployed Percent of the population living below the federal poverty level Percent of the population 25 years and older with less than a high school education Percent of the population 16 years and older who are Hispanic Percent of the population 16 years and older who are non-Hispanic black Percent of the male population Percent of workers 16 years and older that walked or biked to work Percent of workers 16 years and older that commuted to work for longer than 60 min one way
2013 National Center for Health Statistics classification scheme	County rural–urban classification data
2016 Health Resources and Services Administration Area Health Resource Files (AHRF)	Number of primary care physicians per county
County Health Rankings and Roadmaps project	Percent of the population with limited access to healthy foods

Diagnostic criteria for pre-diabetes is either a fasting plasma glucose (FPG) level between 100 mg/dL and 125 mg/dL, a two hour plasma glucose level between 140 mg/dL and 199 mg/dL during an oral glucose tolerance test (OGTT), or glycated hemoglobin (A1C) level between 5.7% and 6.4% (American Diabetes Association, 2019). Pre-diabetes data for 2013 and 2016 were extracted from the Behavioral Risk Factor Surveillance System (BRFSS) database, which was obtained from the Florida Department of Health. The BRFSS is conducted by state health departments, with technical, methodological and financial assistance from the Centers for Disease Control and Prevention (CDC) (Centers for Disease Control and Prevention, 2019). This study used data for 2013 and 2016 because every 3 years, the Florida Department of Health conducts large sampling that allows for county-level estimates to be computed from the BRFSS. At the time this study was conducted, the most recent Florida BRFSS datasets for which these county-level estimates could be computed were the 2013 and 2016 datasets. Although the data collection for 2019 had been completed, the dataset was still under embargo at the time this study was conducted. Pre-diabetes status was based upon self-reports from adult (18 years and older) respondents who reported having been told by a doctor that they had pre-diabetes, unrelated to pregnancy. Additional data obtained for each respondent included county of residence, age, body mass index (BMI), physical activity level, arthritis, disability (defined as an activity limitation due to health problems), and health insurance status. Missing responses and those in which the respondent refused to answer were excluded from the analysis. All data were aggregated to the county level using SAS (Statistical Analysis System (SAS) Version 9.4; SAS Institute. Cary, NC, USA). Pre-diabetes prevalence were age-adjusted to the 2010 United States standard population using the following age groups: 18–44, 45–64, and 65 years and older (Li et al., 2014).

County-level socioeconomic, demographic, and commute data were obtained from the 2012-2016 ACS 5-year estimates (US Census Bureau, 2016a). Socioeconomic characteristics included: median household income, percent of the population 16 years and older who were unemployed, percent of the population living below the federal poverty level, and percent of the population 25 years and older with less than a high school education. Demographic characteristics included percentage of Hispanic population, percentage of non-Hispanic black population, and percentage of male population. Commute data included percent of the population that walked or biked to work and those that commuted to work for longer than 60 min one way.

Rural–urban classification data for each county were obtained from the 2013 National Center for Health Statistics (NCHS) classification scheme (Ingram & Franco, 2014). This classification scheme has a total of six categories within the broad categories of metropolitan or nonmetropolitan (Fig. 1). Metropolitan categories include large, medium and small metro counties. Large metro counties have 1 million residents or more (Ingram & Franco, 2014). Large metro counties are categorized into large central and large fringe metro counties. Medium metro counties have 250,000–999,000 inhabitants, while small metro counties have fewer than 250,000 people (Ingram & Franco, 2014). Nonmetropolitan categories include micropolitan counties, which contain urban cluster populations comprising 10,000–49,999 people, and noncore counties, which are rural areas that do not qualify either as metropolitan or micropolitan counties (Ingram & Franco, 2014).

The number of primary care physicians per county was obtained from the 2016 Area Health Resource Files (AHRF) from the Health Resources and Services Administration (HRSA) (Health Resources and Services Administration, 2020). The 2016 county population estimate was used to calculate the number of primary care physicians per 1,000. The percent of the population with limited access to healthy foods was obtained from the County Health Rankings and Roadmaps project. The percent of the population with limited access to healthy foods was defined based upon annual family income (200% of the federal poverty level or less), and distance from a grocery store (further than 10 miles in rural areas, or one mile in non-rural areas) (University of Wisconsin Population Health Institute, 2019). All data obtained as percentages were converted to proportions for analysis. County-level data were imported in ArcGIS for mapping (ESRI, 2017).

### Descriptive analyses

All descriptive analyses were performed in SAS 9.4 (SAS Institute, 2016). Shapiro–Wilk test was used to assess for normality of distribution of continuous county-level variables. When continuous variables were not normally distributed, medians and interquartile ranges were used to summarize the data, otherwise means and standard deviations were used.

### 2013 and 2016 comparisons

A one-tailed test of equality of proportions was performed in Stata version 15 (StataCorp, College Station, TX, USA) to identify significant changes in pre-diabetes prevalence between 2013 and 2016, using the Stata command prtesti. The Simes method was used to adjust for multiple comparisons. This method was also used to identify differences in county-level characteristics between the two time periods. County level median household incomes between 2013 and 2016 were considered significantly different if their 90% confidence intervals did not overlap.

### Cluster investigation and identification

Tango’s flexible spatial scan statistic (FSSS) was implemented in FleXScan (Tango & Takahashi, 2005) to investigate and identify high-risk spatial clusters of pre-diabetes. The statistic imposes a large number of overlapping, flexibly shaped scanning windows of variable sizes over the study area in order to detect both circular and irregularly shaped clusters, up to a specified maximum size (Tango & Takahashi, 2005). If the scanning window encloses the centroid of a county, that entire county is included in the window. The number of cases within this window are compared with the number of cases that would be expected under the null hypothesis of complete spatial randomness (Tango & Takahashi, 2005).

In this study, the maximum spatial scanning window size was set at 10 counties, specifying binomial probability model. Restricted log-likelihood ratio (LLR) and 999 Monte Carlo replications were used for statistical inference. The most likely clusters were ordered on the basis of the restricted log-likelihood ratios. The primary or most likely cluster was identified as the cluster with the largest value of the restricted log-likelihood ratio. The null hypothesis of complete spatial randomness was rejected when the simulated *p*-value was ≤0.05. Only secondary clusters with a prevalence ratio (PR) greater or equal to 1.2 were reported to avoid reporting clusters with very low risk.

### Assessment of correlations among predictor variables

To avoid multicollinearity during investigation of predictors of pre-diabetes prevalence, Spearman’s rank correlation coefficient was used to identify highly correlated potential predictor variables. Only one of a pair of highly correlated (*r* ≥ 0.7) potential predictors of pre-diabetes prevalence was assessed for potential association with the outcome. The choice of the variable to retain was based on biological and statistical considerations.

### Investigation of predictors of geographic distribution of pre-diabetes

Global multivariable regression modeling was performed using SAS 9.4 (SAS Institute, 2016). The multivariable model with county-level age adjusted pre-diabetes prevalence as outcome was built in two steps. In the first step, univariable associations were assessed using a liberal *p*-value of 0.15. Variables with significant univariable associations were considered for multivariable modeling in step two. During step two, the multivariable model was fit to the data using the generalized linear model procedure in SAS, using manual backwards elimination and a critical *p*-value of ≤0.05 (SAS Institute, 2016). Non-significant variables were considered potential confounders if their removal from the model resulted in a change of greater than 20% in the estimated regression coefficients of any of the remaining variables in the model.

### Cartographic displays

All geographic information system (GIS) manipulations and cartographic displays were performed in ArcGIS (ArcGIS Desktop 10.6.1; ESRI, Redlands, CA, USA). Age-adjusted pre-diabetes prevalence for 2013 and 2016 were displayed in choropleth maps. The critical intervals used in the choropleth maps of 2013 were determined using Jenk’s optimization classification scheme. For consistency and to facilitate comparisons, the intervals used to display the 2013 prevalence data were also used in the choropleth maps for 2016 data.

Significant spatial clusters of high pre-diabetes prevalence were also displayed using ArcGIS. In addition, statistically significant changes in county-level pre-diabetes prevalence estimates between 2013 and 2016 were mapped at the county level using ArcGIS. Significant predictors of pre-diabetes were also displayed in choropleth maps, as were significant changes in these characteristics between 2013 and 2016.

## Results

### Descriptive analyses

A total of 36,955 respondents participated in the 2016 Florida BRFSS Survey of whom 584 had missing age data and were excluded from analysis, leaving 36,371 for analysis. Respondents ranged in age from 18 to 99 years, with a median of 60 and an interquartile range of 45 to 71 years. Among respondents who reported race/ethnicity, the most commonly represented group was non-Hispanic white (57.9%), followed by Hispanic (23.4%), non-Hispanic black (14.1%), and Asian (2.7%). Those who identified as American Indian or Alaska Native represented 0.3% of the population while 1.6% identified as “other” race or ethnicity.

**Figure 2:**
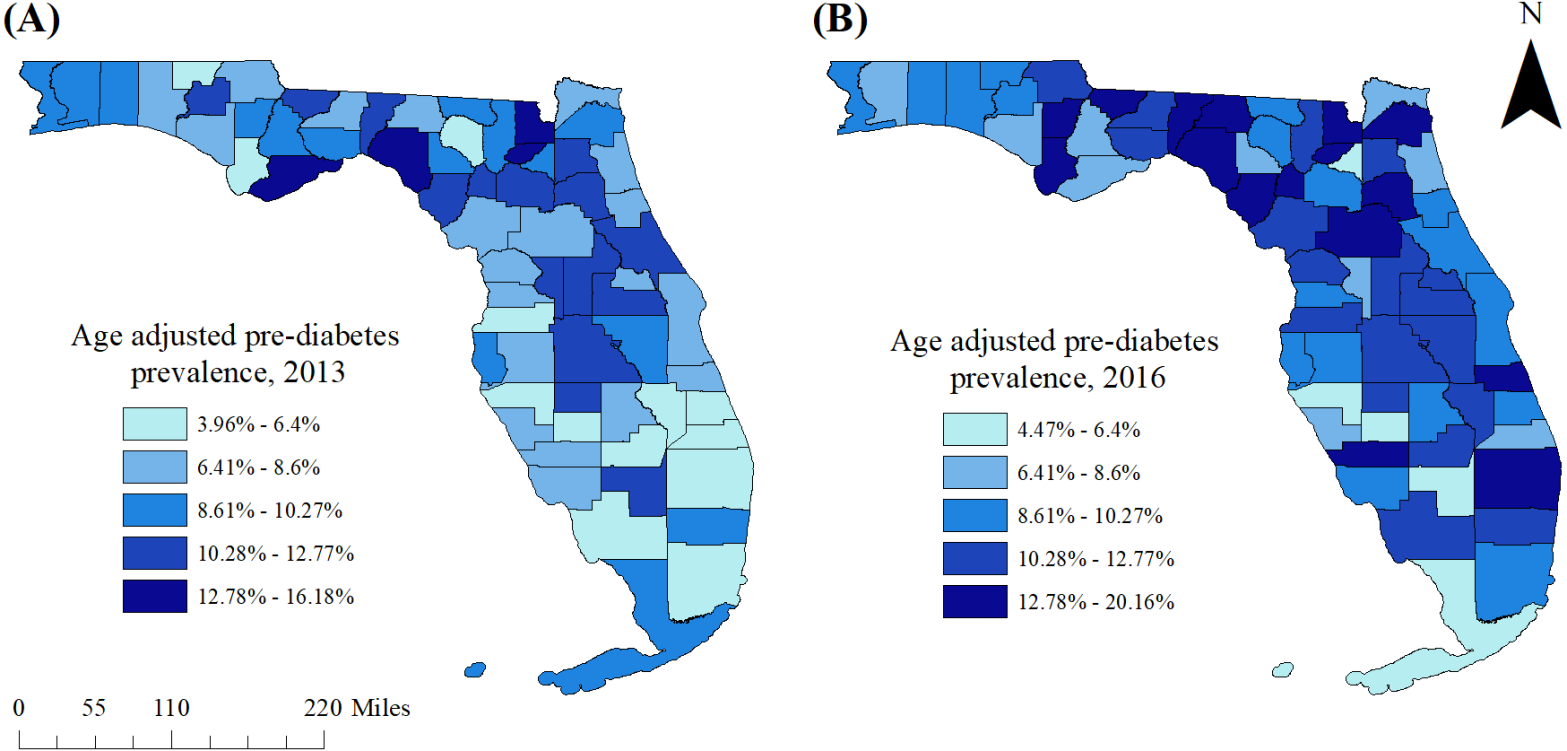
Age-adjusted county-level pre-diabetes prevalence in Florida, (A) 2013 and (B) 2016.

The state-wide age-adjusted pre-diabetes prevalence in 2013 was 8.0%. This increased to 10.5% in 2016, but varied from 4.5% (DeSoto County) to 20.2% (Calhoun County) (Figs. 1, 2A–2B). Overall, more counties had high prevalence proportions of pre-diabetes in 2016 than in 2013. In 2013, counties in the eastern panhandle extending to northern and inland central Florida tended to have high pre-diabetes prevalence. A larger swath of counties with high prevalence proportions in 2016 spanned the central to eastern panhandle and extended south through central Florida. High pre-diabetes prevalence was not limited to rural counties but also occurred in some large central and large fringe metropolitan areas such as the Jacksonville region (Duval County) and Palm Beach County (Figs. 1, 2A–2B).

### Comparison of 2013 and 2016 prevalence estimates

**Figure 3:**
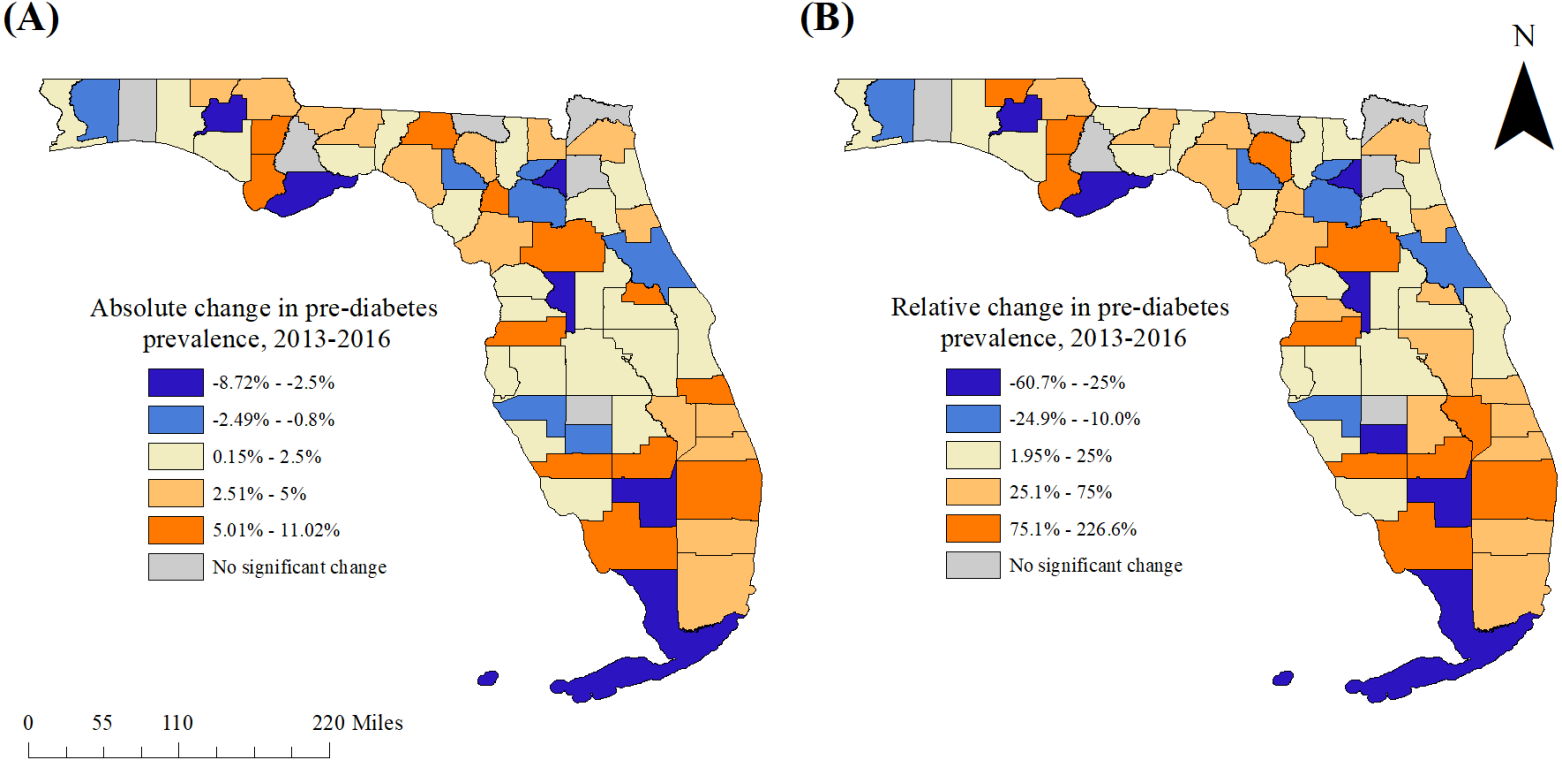
Statistically significant changes in pre-diabetes prevalence in Florida between 2013 and 2016. (A) Absolute change, (B) relative change.

The state-wide age-adjusted prevalence of pre-diabetes increased significantly (*p* < 0.0001) between 2013 and 2016. Significant changes between 2013 and 2016 were identified for all but six counties (Clay, Hamilton, Hardee, Liberty, Nassau, and Okaloosa) (Figs. 1 and 3A–3B). Of the 61 counties with significant changes in pre-diabetes prevalence between 2013 and 2016, significant increases were observed in 78.7% (48/61), while significant decreases were observed in 21.3% (13/61) of the counties. Palm Beach County had the highest relative increase (9.0%, a relative increase of 226.6%), while Hendry County had the highest relative decrease (7.5%, a relative decrease of 60.7%).

### Clusters of pre-diabetes

**Figure 4:**
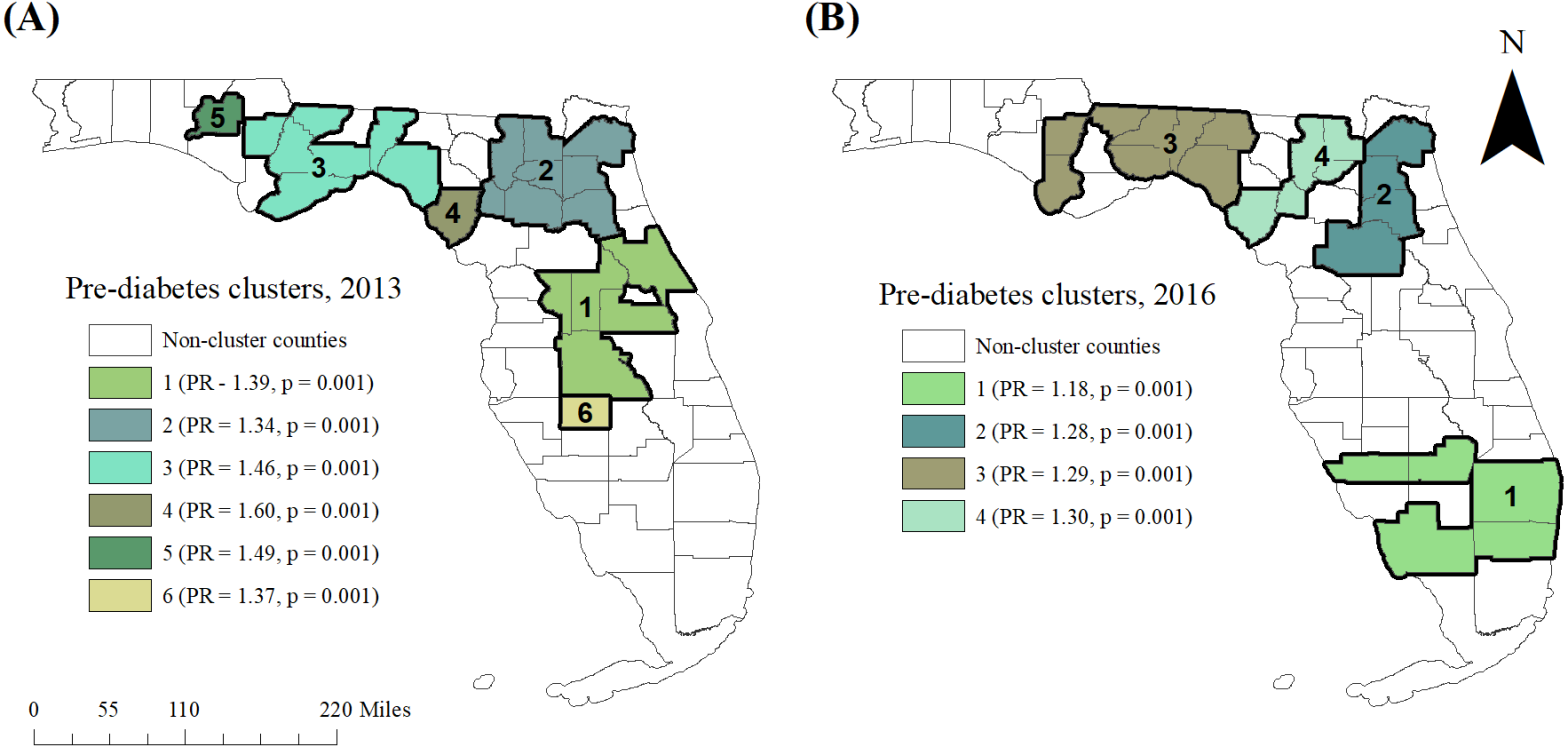
High-risk purely spatial clusters of pre-diabetes in Florida, (A) 2013 and (B) 2016.

Six significant high-prevalence pre-diabetes spatial clusters were identified in 2013, and four were identified in 2016 (Table 2 and Figs. 4A–4B). In 2013, the primary high-prevalence spatial cluster of pre-diabetes consisted of five mostly metropolitan counties in central Florida near Orlando (Lake, Orange, Polk, Sumter, and Volusia counties). In 2016, however, the primary pre-diabetes cluster was located in the southern portion of the state, and included five counties, both rural and metropolitan (Broward, Charlotte, Collier, Glades, and Palm Beach). The prevalence of pre-diabetes in this cluster was 18% higher than that of the state overall (Prevalence Ratio [PR] = 1.18, *p* = 0.001). In contrast, in 2013, some of these southern Florida counties were among those with the lowest prevalence of pre-diabetes in the state. Each of the five counties within the cluster exhibited an increase in pre-diabetes prevalence between 2013 and 2016. Three secondary clusters with prevalence ratios ≥1.2 were identified in 2016, spanning from north-central Florida to the more rural eastern and central panhandle.

**Table 2:** Purely spatial significant clusters of pre-diabetes in Florida, 2013 and 2016.

**Cluster**	**Population**	**Observed cases**	**Counties included**	**PR**^a^	***p*-value**
***2013***					
1	1,803,072	199,681	Lake, Orange, Polk, Sumter, Volusia	1.39	0.001
2	1,042,432	111,467	Alachua, Baker, Bradford, Clay, Columbia, Duval, Gilchrist, Putnam, Union	1.34	0.001
3	99,858	11,650	Calhoun, Franklin, Gadsden, Jefferson, Liberty, Taylor, Wakulla	1.46	0.001
4	12,671	1,617	Dixie	1.60	0.001
5	18,128	2,161	Washington	1.49	0.001
6	13,948	1,523	Hardee	1.37	0.001
***2016***					
1	2,260,022	279700	Broward, Charlotte, Collier, Glades, Palm Beach	1.18	0.001
2	1,014,571	136768	Clay, Duval, Marion, Putnam	1.28	0.001
3	322,559	43629	Calhoun, Gadsden, Gulf, Jefferson, Leon, Madison, Taylor, Wakulla	1.29	0.001
4	112,503	15421	Baker, Columbia, Dixie, Gilchrist, Union	1.30	0.001

**Notes.**

a Prevalence ratio.

### Summary statistics of potential predictors of pre-diabetes

Summary statistics of the investigated potential predictor variables are shown in Table 3. These variables exhibited geographic variation across the state. For instance, counties with low median household income levels were concentrated in three regions: the eastern panhandle, the most rural portion of the state, the central panhandle just west of the Tallahassee region, and inland south-central Florida. Counties with the lowest numbers of primary care physicians per capita overlapped with many of the counties with low median household income levels, and tended to be concentrated in the rural eastern panhandle. Several counties with relatively high proportions of residents without health insurance coverage were also located across the panhandle, as well as inland central Florida and the southernmost portion of the state.

**Table 3:** Summary statistics of potential predictors of county-level pre-diabetes prevalence in Florida, 2016.

**Predictor variable**	**Mean**	**SD**^a^	**Median**	**Min. (county)**	**Max. (county)**	**IQR**^b^
Proportion that walk or bike to work^*^	0.021	0.015	0.019	0.005 (Washington)	0.111 (Monroe)	0.013
Proportion obese	0.313	0.065	0.306	0.143 (Martin)	0.457 (Union)	0.081
Primary care physicians per 1,000 persons^*^	0.548	0.335	0.506	0 (Liberty)	2.076 (Alachua)	0.429
Proportion with less than high-school education^*^	0.135	0.062	0.124	0.044 (St. Johns)	0.326 (Hendry)	0.087
Proportion with arthritis	0.282	0.062	0.290	0.152 (Wakulla)	0.463 (Glades)	0.075
Proportion non-Hispanic black^*^	0.143	0.093	0.117	0.028 (Citrus)	0.536 (Gadsden)	0.104
Proportion Hispanic^*^	0.122	0.120	0.078	0.017 (Holmes)	0.673 (Miami-Dade)	0.109
Median household income (in $10,000)^*^	4.521	0.838	4.422	2.981 (Madison)	6.952 (St. Johns)	1.369
Proportion with a commute >60 min.^*^	0.083	0.035	0.078	0.020 (Hamilton)	0.183 (Bradford)	0.049
Proportion physically inactive^*^	0.326	0.070	0.311	0.211 (Martin)	0.572 (Dixie)	0.102
Proportion without health insurance coverage^*^	0.169	0.048	0.159	0.080 (Sumter)	0.346 (DeSoto)	0.054
Proportion with limited access to healthy foods^*^	0.093	0.057	0.090	0 (Gilchrist, Wakulla)	0.310 (Glades)	0.060
Proportion below the federal poverty line^*^	0.111	0.032	0.108	0.043 (Sumter)	0.204 (DeSoto)	0.036
Proportion reporting a disability	0.236	0.046	0.236	0.127 (Miami-Dade)	0.342 (Levy)	0.064
Proportion unemployed	0.092	0.021	0.087	0.049 (Monroe)	0.150 (Lafayette)	0.026
Proportion male^*^	0.507	0.045	0.488	0.423 (Okeechobee)	0.607 (Franklin)	0.055
NCHS^c^ urban-rural classification^*^	3.746	1.627	3	1	6	2

**Notes.**

a Standard deviation.

b Interquartile range.

c National Center for Health Statistics.

* Non-normally distributed variables.

Overall, 14.1% of the state population was non-Hispanic black, but this also varied across counties. The highest proportions of non-Hispanic black residents tended to be in counties in the central to eastern panhandle along the state’s northern border with Georgia, as well as in counties with urban centers such as the Miami area, Jacksonville, and Tampa.

There was also geographic variation in the unemployment rate, with relatively high levels of unemployment in rural areas such as the inland-south central region as well as the northeastern and north-central panhandle. Higher proportions of the population with limited access to healthy foods also tended to be located in the inland south-central portion of the state, as well as along the central Atlantic coast. Counties with relatively high proportions of the population reporting physical inactivity tended to be located in the rural eastern panhandle and the central panhandle counties just west of the Tallahassee region, as well as in the inland south-central region. Central Florida counties surrounding the Orlando area tended to have high proportions of the population with arthritis, as did several of the rural counties in south-central Florida, while low prevalence proportions of arthritis were observed in Miami-Dade County, the Tallahassee area, and the Jacksonville area.

### Predictors of pre-diabetes

**Figure 5:**
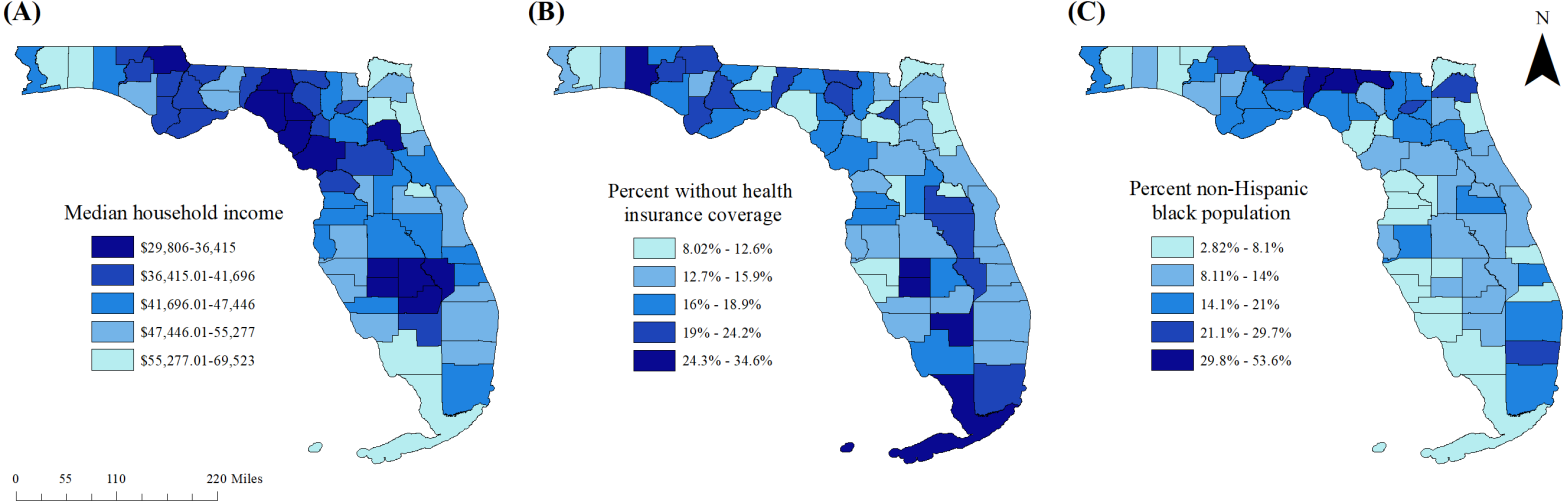
Distribution of significant predictors of county-level pre-diabetes prevalence in Florida, 2016. (A) Median household income, (B) percent without health insurance coverage, (C) percent non-Hispanic black population.

Results of the univariable associations of county-level characteristics with age-adjusted county-level pre-diabetes prevalence are displayed in Table 4 while results of the final multivariable model are shown in Table 5, and the geographic distributions of significant explanatory variables are displayed in Figs. 5A–5C. There were significant negative associations between county pre-diabetes prevalence and median household income (*p* = 0.0113) and proportion of the population without health insurance coverage (*p* = 0.0007), but a significant positive association with proportion of non-Hispanic black population (*p* = 0.0215).

**Table 4:** Univariable associations between county-level characteristics and age-adjusted pre-diabetes prevalence in Florida, 2016.

**Variable**	**β (95% CI**^a^)	*p*-value
Proportion that walk or bike to work	−0.5446 (−1.036, −0.535)	0.030
Proportion obese	0.136 (0.023, 0.250)	0.019
Proportion overweight or obese	0.080 (−0.038, 0.198)	0.185
Primary care physicians per 1000 persons	−0.0086 (−0.031, 0.014)	0.460
Prop. with less than high-school education	0.0059 (−0.118, 0.129)	0.925
Proportion with arthritis	0.0989 (−0.023, 0.221)	0.111
Proportion non-Hispanic black	0.1132 (0.035, 0.191)	0.004
Proportion Hispanic	−0.0628 (−0.125, −0.001)	0.048
Median household income (in $10,000)	−0.0091 (−0.018, −0.0003)	0.044
Proportion with a commute >60 min.	−0.0666 (−0.284, 0.151)	0.547
Proportion physically inactive	0.0678 (−0.040, 0.176)	0.218
Proportion without health insurance coverage	−0.1674 (−0.321, −0.014)	0.033
Proportion with limited access to healthy foods	0.0406 (−0.093, 0.174)	0.550
Proportion under the federal poverty line	−0.0298 (−0.266, 0.207)	0.805
Proportion reporting a disability	0.0371 (−0.128, 0.202)	0.660
Proportion unemployed	0.1449 (−0.226, 0.516)	0.444
Proportion male	−0.0101 (−0.180, 0.160)	0.907
NCHS^b^ urban-rural classification	0.0001(−0.005, 0.005)	0.953

**Notes.**

a Confidence interval.

b National Center for Health Statistics.

**Table 5:** Final multivariable regression model results showing statistically significant predictors of pre-diabetes prevalence at the county level in Florida, 2016.

**Predictor variable**	**β (95% CI**^a^)	**SE**^b^	*χ*^2^	*p*-value
Median household income (in $10,000)	−0.012 (−0.021, −0.003)	0.005	6.41	0.011
Proportion without health insurance coverage	−0.257 (−0.406, −0.108)	0.030	39.4	0.001
Proportion non-Hispanic black	0.088 (0.013, 0.164)	0.038	5.29	0.022

**Notes.**

a Confidence interval.

b Standard error.

**Figure 6:**
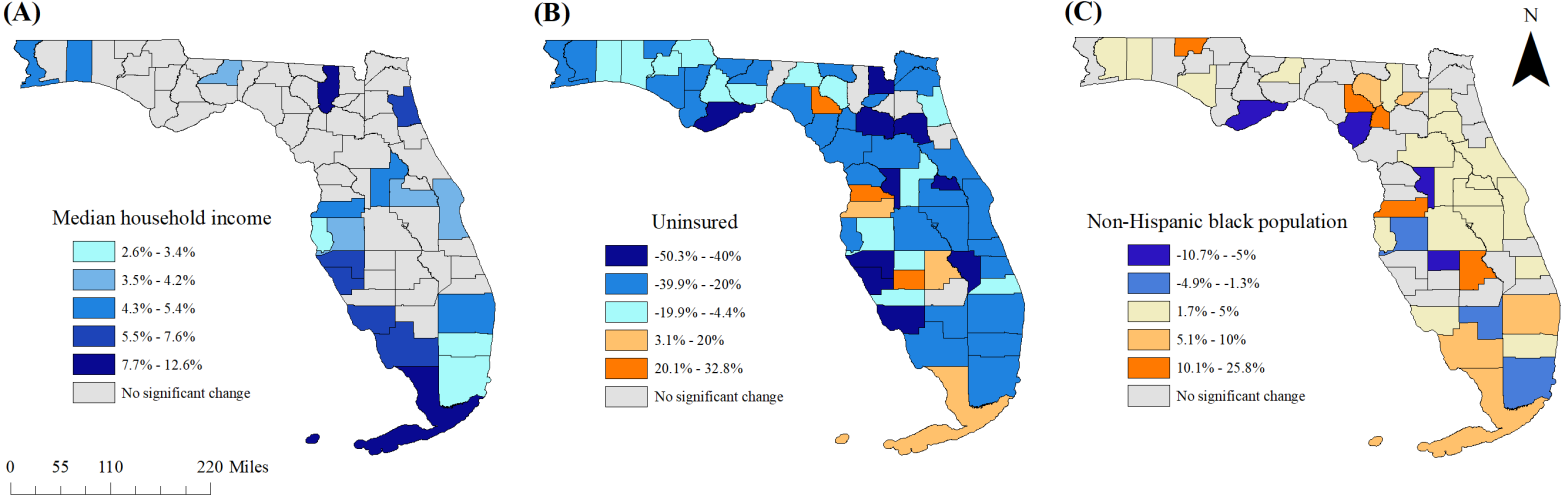
Statistically significant changes in predictors of county-level pre-diabetes prevalence in Florida between 2013 and 2016. (A) Median household income, (B) percent without health insurance coverage, (C) percent non-Hispanic black population

### Changes in county-level characteristics between 2013 and 2016

Relative changes in significant county-level predictor variables between 2013 and 2016 are displayed in Figs. 6A–6C. Significant increases in median household income were observed in 19 counties. None of the counties exhibited statistically significant decreases in median household income. Counties that did not have significant increases in median household income tended to be in more rural areas, frequently overlapping with counties that had high pre-diabetes prevalence as well as increases in prevalence of the condition between 2013 and 2016. Of the 61 counties with significant changes in proportion of the population with health insurance coverage, the majority (90.2%) were decreases in the proportion of the population with coverage.

 Significant changes in the proportion of non-Hispanic black population between 2013 and 2016 occurred in 36 (53.7%) counties, with many of these counties exhibiting a relative change of less than 5%. The proportion of non-Hispanic black population increased in most of the counties surrounding Orlando, but otherwise there were no clear geographic patterns or obvious overlap with changes in pre-diabetes prevalence.

## Discussion

This study investigated county-level geographic disparities of pre-diabetes prevalence in Florida, identified predictors of the observed disparities as well as changes in disease burden between 2013 and 2016. The findings are useful for guiding the allocation of resources for the continued implementation of targeted prevention and control programs.

### Spatial patterns and clusters of pre-diabetes prevalence

The results indicated that geographic disparities in pre-diabetes prevalence continue to exist in Florida. While previous studies have investigated the geographic distribution of this condition, few have used rigorous spatial statistical/epidemiological techniques to investigate and identify these disparities. To best inform needs-based health planning, there is need for continuous monitoring and rigorous assessments to characterize the distribution of pre-diabetes and identify predictors of high-risk areas so as to target intervention programs. The existence of spatial disparities of pre-diabetes prevalence observed in this study is consistent with findings from the Reasons for Geographic and Racial Differences in Stroke (REGARDS) study, which reported that the odds of pre-diabetes among adults ≥45 years old living in the “stroke belt” were higher than for those living outside this region (Barker et al., 2011; Lee et al., 2014).

Spatial clustering of county-level pre-diabetes prevalence has previously been described. However, no rigorous epidemiological approaches have been consistently used in these investigations. The current study mitigates this problem by using Tango’s flexible spatial scan statistic (FSSS) which is a rigorous approach that is robust and does not have the problem of multiple comparisons that other methods such as Moran’s Local Indicators of Spatial Association (LISA) do have. Moreover, application of this approach can be scaled to other states to provide useful information to guide evidence-based health planning. Additionally, Tango’s FSSS identifies clusters without the need for pre-specification of the suspected cluster location or size and thus eliminates pre-selection bias. An additional advantage of using Tango’s FSSS is the flexibly shaped scanning window, which has high power for the detection of clusters that are not circular in shape (Tango & Takahashi, 2005).

It is worth pointing out that other studies have used FSSS to identify spatial clusters of other health outcomes such as measles (Tang et al., 2017), cancer (Katayama et al., 2014; Amin, Nelson & McDougall, 2018), suicide mortality (Yamaoka et al., 2020), and cardiovascular diseases (Roberson et al., 2019; Odoi et al., 2019). Recent research in Florida has demonstrated that FSSS is a useful technique for the identification of geographic disparities in stroke prevalence (Roberson et al., 2019) and hospitalizations due to myocardial infarction (Odoi et al., 2019). Suffice it to say that populations living within the identified pre-diabetes clusters identified in the current study have disproportionately high risks of the condition and, therefore, control efforts should focus on these areas in order to reduce the observed disparities.

### Predictors of pre-diabetes

Pre-diabetes awareness reportedly varies with factors such as age, level of educational attainment, health insurance coverage, and food security (Li et al., 2013; Ding et al., 2014). Data from the National Health and Nutrition Examination Survey (NHANES) indicated that while the condition is common among American adults, with approximately one-third estimated to have pre-diabetes, only 11% were aware that they had the condition (Li et al., 2013). Detection of those at high risk is important so that interventions aimed at preventing the progression of pre-diabetes to diabetes may be pursued. The fact that the proportion of the population without health insurance coverage was negatively associated with pre-diabetes prevalence may, to some extent, suggests that these prevalence estimates might be proxy measures of access to healthcare services. Indeed, several counties to the west of the Tallahassee region in the panhandle that had relatively high rates of un-insurance, were part of a high-prevalence diabetes cluster, but were not part of a pre-diabetes cluster.

The significant positive associations observed between county pre-diabetes prevalence and the proportion of the population that was non-Hispanic black suggests that the geographic disparities in the prevalence of the conditions in Florida are at least partly attributable to racial disparities. Significantly higher odds of pre-diabetes have been reported among black participants in the REGARDS study in comparison to the white participants, a finding which changed only minimally after adjusting for region (Lee et al., 2014).

Median household income was a significant predictor of county pre-diabetes prevalence. Economic stability is a key social determinant of health (Office of Disease Prevention and Health Promotion, 2019). It is known that income affects access to physical activity opportunities as well as healthy foods which have been shown to impact development of pre-diabetes and potential progression to diabetes. Thus, healthy eating habits are an important aspect of pre-diabetes prevention and management and may be influenced by purchasing power and accessibility of nutritious foods. Therefore, county and local food environments may represent potential targets for policies and interventions, in addition to individual-level programs.

### Changes in pre-diabetes prevalence between 2013 and 2016

There was an increase in the statewide prevalence of pre-diabetes as well as among the majority of counties between 2013 and 2016. However, since prevalence estimates were based upon self-reports, it is difficult to determine the extent to which the observed increases are attributable to true increases within the population rather than changes in diagnostic and reporting practices. However, the overlap in the spatial patterns of improved health insurance coverage and increased pre-diabetes prevalence may suggest that these increases might, in part, be due to increased awareness and reporting of the condition. Improved diagnosis and reporting may also account for some of the observed increases in pre-diabetes prevalence.

Temporal changes were also observed in some county-level characteristics that may have contributed to the changes in pre-diabetes prevalence during the study period. The findings suggest that areas with indicators of economic stagnation were more likely to have increases in pre-diabetes prevalence during the study period. For example, the rural counties in the panhandle and south-central Florida tended not to have significant changes in median household income. The observed temporal changes warrant ongoing monitoring of the conditions within Florida. Additionally, county characteristics exhibited changes that varied from county to county across the state, further emphasizing the importance of evidence-based planning that recognizes local differences in population characteristics, disease burden and health needs.

### Strengths and weaknesses

This study has used rigorous spatial epidemiological tools to investigate pre-diabetes disparities and predictors in Florida. Continued use of such approaches is crucial for guiding evidence-based health planning and service provision. Understanding the local and regional changes in health conditions is important for guiding the targeting of control efforts to improve help outcomes for all.

Due to the nature of BRFSS data, pre-diabetes status of respondents was based upon self-reports and therefore, the exact diagnostic criteria used were not available. The use of differing definitions of the condition or methodological changes may impact prevalence estimates at the population level (Selvin et al., 2014), and may limit the ability to make accurate temporal comparisons. Limited awareness of pre-diabetes among respondents may have resulted in underestimates of the true burden of the conditions. Other studies have reported that awareness of pre-diabetes varies based on demographic characteristics (Li et al., 2013; Selvin et al., 2014). Presumably, awareness of pre-diabetes status may vary between counties, but the extent to which this occurs is unknown.

## Conclusions

This study confirmed the persistence of geographic disparities in the prevalence of diagnosed pre-diabetes at the county level in Florida. It demonstrated the usefulness of Tango’s flexible scan statistic for identifying high prevalence clusters of the condition. The study also showed an overall, state-wide increase in pre-diabetes prevalence in Florida. Counties with stagnant median income levels tended to have temporal increases in pre-diabetes prevalence, highlighting the importance of place-based factors in chronic disease risk management. Continued monitoring of pre-diabetes distribution is warranted, with careful attention to factors such as healthcare access and patient awareness of the condition, which may affect reporting. The identification of high-prevalence clusters of pre-diabetes with flexible scan statistics is useful for identifying populations at greatest risk, and informing the allocation of resources within the state. Ongoing monitoring and epidemiologic analyses are important for identifying trends at county and state levels and for identifying factors associated with pre-diabetes that may represent potential targets for health planning and interventions.

##  Supplemental Information

10.7717/peerj.10443/supp-1 Supplemental Information 1 Raw Prediabetes Study Data

10.7717/peerj.10443/supp-2 Supplemental Information 2 Data Dictionary for Prediabetes Study Data

## Additional Information and Declarations

### Data Availability

The following information was supplied regarding data availability: The raw study data and data dictionary are available as Supplemental Files.
