# Supplementary material for: Geographic disparities, determinants, and temporal changes in the prevalence of pre-diabetes in Florida
Source: PeerJ. 2021 Jan 13;9:e10443. doi: 10.7717/peerj.10443 (PMC7811289; doi:10.7717/peerj.10443)
Supplement: Supplemental Information 2 [file peerj-09-10443-s002.docx]

| **Variable Name** | **Variable** |
| --- | --- |
| CountyName | County name |
| Arth_pro16 | Proportion of county adults with arthritis (2016) |
| Inact_pro16 | Proportion of county adults who are physically inactive (2016) |
| Unins_pro16 | Proportion of county adults without health insurance coverage (2016) |
| Pop16over_16 | County residents over the age of 16 (2016) |
| Pop16over_13 | County residents over the age of 16 (2013) |
| MedHHinc10k_13 | Median household income (in $10,000) (2013) |
| Nonhispbl13 | Proportion of county residents 16 and older who are non-Hispanic Black (2013) |
| Arth_pro13 | Proportion of county adults with arthritis (2013) |
| Inact_pro13 | Proportion of county adults who are physically inactive (2013) |
| Unins_pro13 | Proportion of county adults without health insurance coverage (2013) |
| Nonhispbl16 | Proportion of county residents 16 and older who are non-Hispanic Black (2016) |
| Unemploy16 | Proportion of county residents 16 and older who are unemployed (2016) |
| Unemploy13 | Proportion of county residents 16 and over who are unemployed (2013) |
| NCHS_class | NCHS rural-urban categorization |
| Meantrav | Mean travel time to work for county workers 16 and older (2016) |
| Walkbike | Proportion of county workers 16 and older that walk or bike to work (2016) |
| Over60com | Proportion of county workers 16 and older with commute > 60 minutes (2016) |
| Ov_Ob | Proportion of county adults who are overweight or obese (2016) |
| Obes_pro | Proportion of county adults who are obese (2016) |
| Disab_pro | Proportion of county adults with a disability (2016) |
| LIMACC | Proportion of county residents with limited access to healthy foods |
| Poverty | Proportion of county residents 16 and older under the federal poverty line (2016) |
| Educ_nohs | Proportion of county residents 20-64 with less than a high school education (2016) |
| Hispanic | Proportion of county residents 16 and older who are Hispanic (2016) |
| PropMale | Proportion of male county residents (2016) |
| PCP1k | Primary care physicians per 1,000 population (2016) |
| Medhhinc10k_16 | Median household income (in $10,000) (2016) |
| Prediab_std | Age-standardized county prediabetes prevalence (2016) |
| Agestdpre13 | Age-standardized county prediabetes prevalence (2013) |
